# Disordered structure of propane-1,2-diaminium dichloride

**DOI:** 10.1107/S1600536811001036

**Published:** 2011-01-12

**Authors:** Izabela Pospieszna-Markiewicz, Ewa Zielaskiewicz, Wanda Radecka-Paryzek, Maciej Kubicki

**Affiliations:** a Department of Chemistry, Adam Mickiewicz University, Grunwaldzka 6, 60-780 Poznań, Poland

## Abstract

In the title compound, C_3_H_12_N_2_^2+^·2Cl^−^, the cations are disordered over two well resolved positions in a 0.525 (13):0.475 (13) ratio. The disorder involves two C atoms which assume positions that make an almost mirror-sym­metrical system. Similar disorder is observed both at room temperature and at 120 (1) K. The conformation of the NCCN chain in both components is close to *trans* (the torsion angles *ca* ±170°), while that of CCCN chain is close to *gauche* (±50°). In the crystal, a network of relatively strong N—H⋯Cl hydrogen bonds connects the cations and anions into one-cation-deep layers parallel to (001); there are *R*_2_^4^(8) and *R*_2_^4^(11) ring motifs within the plane. The planes are only loosely connected by van der Waals contacts and electrostatic inter­actions between cations and anions.

## Related literature

For general literature on polyamines, see, for example: Hosseinkhani *et al.* (2004 ▶); Pospieszna-Markiewicz *et al.* (2006 ▶, 2007 ▶); Ziebarth & Wang (2009 ▶); Itaka *et al.* (2010 ▶). For the crystal structures of simple salts of propane-1,2-diaminium, see: Aghabozorg *et al.* (2008 ▶); Gerrard & Weller (2002 ▶); Lee & Harrison (2003 ▶); Todd & Harrison (2005 ▶). 
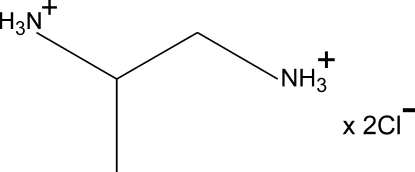


## Experimental

### 

#### Crystal data

C_3_H_12_N_2_^2+^·2Cl^−^
*M* *_r_* = 147.05
Orthorhombic, 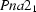

*a* = 10.985 (3) Å
*b* = 7.079 (2) Å
*c* = 9.698 (2) Å
*V* = 754.1 (3) Å^3^
*Z* = 4
Cu *K*α radiation
μ = 6.95 mm^−1^
*T* = 120 K
0.25 × 0.1 × 0.05 mm

#### Data collection

Oxford Diffraction Xcalibur Eos diffractometer
Absorption correction: multi-scan (*CrysAlis PRO*; Oxford Diffraction, 2009 ▶) *T*_min_ = 0.640, *T*_max_ = 1.000
2817 measured reflections
1306 independent reflections
1265 reflections with *I* > 2σ(*I*)
*R*_int_ = 0.032

#### Refinement

*R*[*F* ^2^ > 2σ(*F* ^2^)] = 0.034
*wR*(*F* ^2^) = 0.098
*S* = 1.13
1306 reflections
86 parameters
1 restraint
H-atom parameters constrained
Δρ_max_ = 0.33 e Å^−3^
Δρ_min_ = −0.34 e Å^−3^
Absolute structure: Flack (1983 ▶), 473 Friedel pairs
Flack parameter: 0.09 (3)

### 

Data collection: *CrysAlis PRO* (Oxford Diffraction, 2009 ▶); cell refinement: *CrysAlis PRO*; data reduction: *CrysAlis PRO*; program(s) used to solve structure: *SIR92* (Altomare *et al.*, 1993 ▶); program(s) used to refine structure: *SHELXL97* (Sheldrick, 2008 ▶); molecular graphics: *SHELXTL* (Sheldrick, 2008 ▶) and *Mercury* (Macrae *et al.*, 2008 ▶); software used to prepare material for publication: *SHELXL97*.

## Supplementary Material

Crystal structure: contains datablocks I, global. DOI: 10.1107/S1600536811001036/cv5036sup1.cif

Structure factors: contains datablocks I. DOI: 10.1107/S1600536811001036/cv5036Isup2.hkl

Additional supplementary materials:  crystallographic information; 3D view; checkCIF report

## Figures and Tables

**Table 1:** Hydrogen-bond geometry (Å, °)

*D*—H⋯*A*	*D*—H	H⋯*A*	*D*⋯*A*	*D*—H⋯*A*
N1—H1*D*⋯Cl1^i^	0.91	2.25	3.161 (3)	175
N1—H1*E*⋯Cl2^ii^	0.91	2.37	3.192 (3)	151
N1—H1*F*⋯Cl2	0.91	2.31	3.187 (3)	161
N4—H4*B*⋯Cl1	0.91	2.42	3.186 (3)	142
N4—H4*A*⋯Cl1^iii^	0.91	2.27	3.136 (3)	160
N4—H4*C*⋯Cl2^iv^	0.91	2.21	3.123 (2)	178

Symmetry codes: (i) 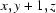; (ii) 
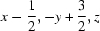
; (iii) 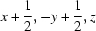; (iv) 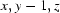.

**Fig. 1.**
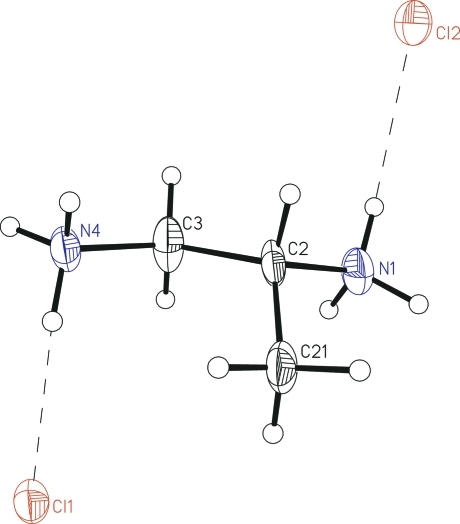
Anisotropic ellipsoid representation of I together with atom labelling scheme. The ellipsoids are drawn at 50% probability level, hydrogen atoms are depicted as spheres with arbitrary radii; hydrogen bonds are shown as dashed lines. Only major part of the disordered cation is shown.

**Fig. 2.**
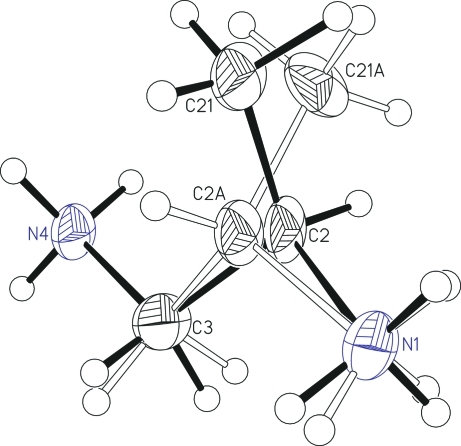
The disordered cation in I: the major part is drawn with solid lines, the minor one - with open lines.

**Fig. 3.**
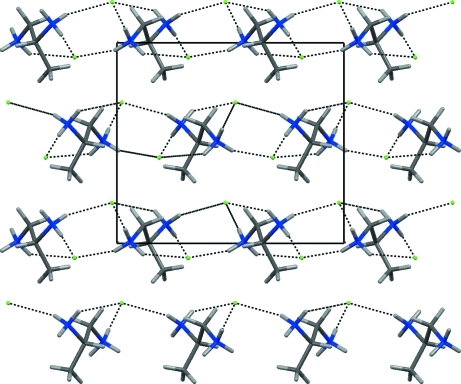
The single layer as seen along direction [100]. Only the cations of major part are shown. Hydrogen bonds are depicted as dashed lines.

**Fig. 4.**
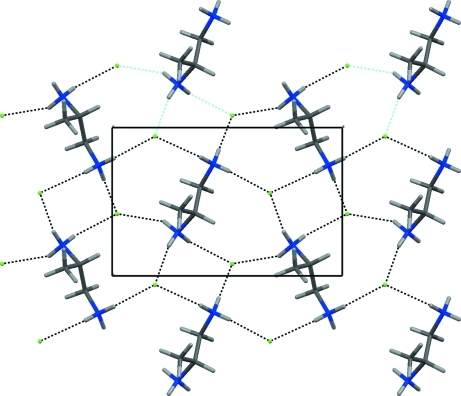
The crystal packing as seen along direction [001]. Only the cations of major part are shown. Hydrogen bonds are depicted as dashed lines.

## supplementary crystallographic information

### Comment

The study of polycation structure, counterions and the nature of the interaction between low-molecular-weight salts with some electrolites in solutions are very important to understanding behaviour biogenic polyamines under normal physiological conditions. Aliphatic biogenic polyamines in biological systems exist as polycations which interact with nucleic acid polyanions. The crystal structure of the salts of these amines are therefore essential in the modeling of nucleic acids. Complexes formed from DNA and polycations containing primary amine grups are relevant because of their potential use in gene therapy, modifying the conformation and the state of aggregation of DNA (Hosseinkhani *et al.*, 2004; Ziebarth *et al.*, 2009; Itaka *et al.*, 2010). During our study on metal promoted synthesis ofSchiff base complexes derived from various polyamines and salicylaldehyde we isolated the crystals of some salts of polyamines (Pospieszna-Markiewicz *et al.*, 2006, 2007). Here we report another salt, propane-1,2-diaminium dichloride (I, Scheme 1).

It turned out that in the crystal structure the cation is heavily disordered - disorder involves two carbon atoms - between the two positions with the site occupation factors of 0.525 (13) and 0.475 (13). Both alternative positions refined quite well (anisotropically) without any kind of restraints. Fig. 1 shows one of the alternatives (most occupied) and Fig. 2 - the comparison of both disordered cations. Similar disorder is observed at room temperature with s.o.f.'s of 0.57 (4) and 0.43 (4), so this disorder is rather of statistical nature.

Both alternative cations have opposite signs of torsion angles. The conformation of N1—C2—C3—N4 chain is extended (torsion angles are 172.0 (4)° and -168.5 (4)° for more and less occupied part, respectively) while the C21—C2—C3—N4 torsion angles are 50.0 (10)° and -47.0 (11)°. Such conformation - we will call it *tg* - is the most popular among the simple propane-1,2-diaminium salts (for instance it was found in the structures of hydrogenarsenate (Todd & Harrison, 2005), bis(6-carboxypyridine-2-carboxylate) (Aghabozorg *et al.*, 2008), or tetrafluoro-beryllium (Gerrard & Weller, 2002). The other possibilities are also observed, for instance in some simple hydrates (*e.g.* arsenate monohydrate, Lee & Harrison, 2003) the *g^+^g^-^* combination is also reported.

In the crystal structure the strong N—H···Cl hydrogen bond connects molecules into two-dimensional, one-molecule deep layers parallel to (001) plane (Fig. 3). The motifs formed can be described, using graph set notations, as rings *R*_2_^4^(8) and *R*_2_^4^(11). It might be noted that for both alternatives the hydrogen atoms involved in these interactions are practically in the same positions. Each chloride anion accepts three hydrogen bonds, in flattened trigonal pyramid coordination. The layers in turn are loosely connected probably by electrostatic interactions between the charged species (Fig. 4).

### Experimental

To a methanol solution (10 ml) of salicylaldehyde (0.043 ml, 0.4 mmol) a methanol solution (10 ml) of 1,2-diaminepropane (0.017 ml, 0.2 mmol) was added dropwise with stirring. After 5 minutes a methanol solution (20 ml) of ErCl_3_.6H_2_O (0.0764 g, 0.2 mmol) was added. The reaction was carried out at room temperature for 75 minutes. The solution volume was than reduced to 10 ml by roto-evaporation and after 7–14 days of slow diffusion of THF into the solution at 6 °C white crystals suitable for X-ray were formed.

### Refinement

Hydrogen atoms were located geometrically (*C*(methyl)-H 0.98 Å, C2—H 1.00 Å, C3—H 0.99 Å, N—H 0.91 Å) and refined in a riding model approximation; the *U*_iso_ values of H atoms were set at 1.2 (1.5 for CH_3_ and NH_3_ groups) times *U*_eq_ of their carrier atom.

### Crystal data

**Table:**

C_3_H_12_N_2_^2+^·2Cl^−^	*F*(000) = 312
*M**_r_* = 147.05	*D*_x_ = 1.295 Mg m^−^^3^
Orthorhombic, *P**n**a*2_1_	Cu *K*α radiation, λ = 1.54178 Å
Hall symbol: P 2c -2n	Cell parameters from 2191 reflections
*a* = 10.985 (3) Å	θ = 4.0–75.5°
*b* = 7.079 (2) Å	µ = 6.95 mm^−^^1^
*c* = 9.698 (2) Å	*T* = 120 K
*V* = 754.1 (3) Å^3^	Block, colourless
*Z* = 4	0.25 × 0.1 × 0.05 mm

### Data collection

**Table:**

Oxford Diffraction Xcalibur Eos diffractometer	1306 independent reflections
Radiation source: Enhance (Mo) X-ray Source	1265 reflections with *I* > 2σ(*I*)
graphite	*R*_int_ = 0.032
Detector resolution: 16.1544 pixels mm^-1^	θ_max_ = 75.7°, θ_min_ = 7.4°
ω scans	*h* = −12→13
Absorption correction: multi-scan (*CrysAlis PRO*; Oxford Diffraction, 2009)	*k* = −8→8
*T*_min_ = 0.640, *T*_max_ = 1.000	*l* = −11→12
2817 measured reflections	

### Refinement

**Table:**

Refinement on *F*^2^	Secondary atom site location: difference Fourier map
Least-squares matrix: full	Hydrogen site location: inferred from neighbouring sites
*R*[*F*^2^ > 2σ(*F*^2^)] = 0.034	H-atom parameters constrained
*wR*(*F*^2^) = 0.098	*w* = 1/[σ^2^(*F*_o_^2^) + (0.0669*P*)^2^ + 0.1755*P*] where *P* = (*F*_o_^2^ + 2*F*_c_^2^)/3
*S* = 1.13	(Δ/σ)_max_ = 0.001
1306 reflections	Δρ_max_ = 0.33 e Å^−^^3^
86 parameters	Δρ_min_ = −0.34 e Å^−^^3^
1 restraint	Absolute structure: Flack (1983), 473 Friedel pairs
Primary atom site location: structure-invariant direct methods	Flack parameter: 0.09 (3)

### Special details

**Table:**

Geometry. All e.s.d.'s (except the e.s.d. in the dihedral angle between two l.s. planes) are estimated using the full covariance matrix. The cell e.s.d.'s are taken into account individually in the estimation of e.s.d.'s in distances, angles and torsion angles; correlations between e.s.d.'s in cell parameters are only used when they are defined by crystal symmetry. An approximate (isotropic) treatment of cell e.s.d.'s is used for estimating e.s.d.'s involving l.s. planes.
Refinement. Refinement of *F*^2^ against ALL reflections. The weighted *R*-factor *wR* and goodness of fit *S* are based on *F*^2^, conventional *R*-factors *R* are based on *F*, with *F* set to zero for negative *F*^2^. The threshold expression of *F*^2^ > σ(*F*^2^) is used only for calculating *R*-factors(gt) *etc*. and is not relevant to the choice of reflections for refinement. *R*-factors based on *F*^2^ are statistically about twice as large as those based on *F*, and *R*- factors based on ALL data will be even larger.

### Fractional atomic coordinates and isotropic or equivalent isotropic displacement parameters (Å^2^)

**Table:**

	*x*	*y*	*z*	*U*_iso_*/*U*_eq_	Occ. (<1)
N1	0.2839 (2)	0.7068 (3)	0.5952 (3)	0.0290 (5)	
H1A	0.2462	0.7953	0.5422	0.043*	0.525 (13)
H1B	0.2269	0.6348	0.6386	0.043*	0.525 (13)
H1C	0.3313	0.7653	0.6592	0.043*	0.525 (13)
H1D	0.2547	0.8030	0.5424	0.043*	0.475 (13)
H1E	0.2252	0.6685	0.6552	0.043*	0.475 (13)
H1F	0.3501	0.7474	0.6432	0.043*	0.475 (13)
C2	0.3589 (9)	0.5771 (12)	0.5034 (11)	0.027 (2)	0.525 (13)
H2	0.4390	0.6396	0.4858	0.032*	0.525 (13)
C21	0.2954 (9)	0.5541 (8)	0.3682 (7)	0.034 (2)	0.525 (13)
H21A	0.2133	0.5043	0.3838	0.051*	0.525 (13)
H21B	0.3412	0.4660	0.3101	0.051*	0.525 (13)
H21C	0.2899	0.6769	0.3219	0.051*	0.525 (13)
C2A	0.3142 (10)	0.5396 (15)	0.5026 (12)	0.027 (2)	0.475 (13)
H2A	0.2358	0.4795	0.4737	0.032*	0.475 (13)
C21A	0.3753 (8)	0.6147 (11)	0.3760 (8)	0.035 (2)	0.475 (13)
H21D	0.4024	0.5091	0.3184	0.052*	0.475 (13)
H21E	0.4458	0.6913	0.4028	0.052*	0.475 (13)
H21F	0.3178	0.6929	0.3241	0.052*	0.475 (13)
C3	0.3821 (3)	0.4007 (4)	0.5893 (4)	0.0328 (7)	
H3A	0.3024	0.3566	0.6246	0.039*	0.525 (13)
H3B	0.4341	0.4308	0.6697	0.039*	0.525 (13)
H3C	0.4454	0.4649	0.6448	0.039*	0.475 (13)
H3D	0.3222	0.3452	0.6540	0.039*	0.475 (13)
N4	0.4396 (2)	0.2468 (3)	0.5090 (3)	0.0253 (5)	
H4A	0.5061	0.2925	0.4640	0.038*	
H4B	0.3854	0.2007	0.4466	0.038*	
H4C	0.4629	0.1526	0.5672	0.038*	
Cl1	0.18604 (6)	0.05885 (8)	0.42805 (6)	0.0279 (2)	
Cl2	0.52155 (6)	0.91848 (8)	0.70308 (9)	0.0328 (2)	

### Atomic displacement parameters (Å^2^)

**Table:**

	*U*^11^	*U*^22^	*U*^33^	*U*^12^	*U*^13^	*U*^23^
N1	0.0357 (13)	0.0183 (12)	0.0329 (12)	0.0056 (9)	0.0017 (10)	−0.0040 (9)
C2	0.036 (5)	0.009 (4)	0.035 (4)	0.005 (3)	−0.003 (4)	0.000 (3)
C21	0.053 (5)	0.015 (3)	0.034 (4)	0.007 (3)	−0.014 (3)	0.002 (2)
C2A	0.033 (5)	0.014 (5)	0.033 (4)	0.004 (4)	0.009 (4)	−0.002 (3)
C21A	0.049 (5)	0.022 (3)	0.033 (3)	0.015 (3)	0.008 (3)	0.011 (3)
C3	0.0530 (18)	0.0185 (13)	0.0269 (14)	0.0108 (12)	0.0001 (13)	0.0005 (10)
N4	0.0319 (12)	0.0141 (11)	0.0298 (12)	0.0028 (9)	−0.0006 (9)	−0.0012 (8)
Cl1	0.0323 (3)	0.0199 (3)	0.0314 (3)	0.0026 (2)	−0.0011 (3)	−0.0003 (2)
Cl2	0.0350 (4)	0.0252 (3)	0.0383 (4)	−0.0001 (2)	−0.0050 (3)	0.0103 (3)

### Geometric parameters (Å, °)

**Table:**

N1—C2	1.522 (10)	C2A—C3	1.493 (12)
N1—C2A	1.523 (10)	C2A—C21A	1.496 (14)
N1—H1A	0.9100	C2A—H2A	1.0000
N1—H1B	0.9100	C21A—H21D	0.9800
N1—H1C	0.9101	C21A—H21E	0.9800
N1—H1D	0.9101	C21A—H21F	0.9800
N1—H1E	0.9100	C3—N4	1.481 (4)
N1—H1F	0.9100	C3—H3A	0.9900
C2—C21	1.495 (13)	C3—H3B	0.9900
C2—C3	1.523 (10)	C3—H3C	0.9900
C2—H2	1.0000	C3—H3D	0.9899
C21—H21A	0.9800	N4—H4A	0.9100
C21—H21B	0.9800	N4—H4B	0.9100
C21—H21C	0.9800	N4—H4C	0.9100
			
C2—N1—H1A	109.3	C2A—C21A—H21D	109.5
C2A—N1—H1A	107.5	C2A—C21A—H21E	109.5
C2—N1—H1B	107.7	H21D—C21A—H21E	109.5
C2A—N1—H1B	89.3	C2A—C21A—H21F	109.5
H1A—N1—H1B	109.5	H21D—C21A—H21F	109.5
C2—N1—H1C	111.3	H21E—C21A—H21F	109.5
C2A—N1—H1C	129.1	N4—C3—C2A	113.7 (5)
H1A—N1—H1C	109.5	N4—C3—C2	112.8 (4)
H1B—N1—H1C	109.5	N4—C3—H3A	109.1
C2A—N1—H1D	109.0	C2A—C3—H3A	87.7
C2A—N1—H1E	107.5	C2—C3—H3A	107.4
H1D—N1—H1E	109.5	N4—C3—H3B	109.0
C2A—N1—H1F	111.9	C2A—C3—H3B	126.1
H1D—N1—H1F	109.5	C2—C3—H3B	110.5
H1E—N1—H1F	109.5	H3A—C3—H3B	107.8
C21—C2—N1	109.0 (7)	N4—C3—H3C	108.8
C21—C2—C3	118.0 (7)	C2A—C3—H3C	110.7
N1—C2—C3	105.4 (7)	N4—C3—H3D	109.0
C21—C2—H2	108.0	C2A—C3—H3D	106.6
N1—C2—H2	108.0	H3C—C3—H3D	107.7
C3—C2—H2	108.0	C3—N4—H4A	109.5
C3—C2A—C21A	118.2 (8)	C3—N4—H4B	109.5
C3—C2A—N1	106.8 (7)	H4A—N4—H4B	109.5
C21A—C2A—N1	107.8 (8)	C3—N4—H4C	109.5
C3—C2A—H2A	107.9	H4A—N4—H4C	109.5
C21A—C2A—H2A	107.9	H4B—N4—H4C	109.5
N1—C2A—H2A	107.9		
			
C2A—N1—C2—C21	54.9 (18)	C21A—C2A—C3—C2	45.2 (16)
C2A—N1—C2—C3	−72.6 (19)	N1—C2A—C3—C2	−76.3 (19)
C2—N1—C2A—C3	78.5 (19)	C21—C2—C3—N4	50.0 (10)
C2—N1—C2A—C21A	−49.4 (17)	N1—C2—C3—N4	172.0 (4)
C21A—C2A—C3—N4	−47.0 (11)	C21—C2—C3—C2A	−47.0 (17)
N1—C2A—C3—N4	−168.5 (4)	N1—C2—C3—C2A	74.9 (18)

### Hydrogen-bond geometry (Å, °)

**Table:**

*D*—H···*A*	*D*—H	H···*A*	*D*···*A*	*D*—H···*A*
N1—H1D···Cl1^i^	0.91	2.25	3.161 (3)	175
N1—H1E···Cl2^ii^	0.91	2.37	3.192 (3)	151
N1—H1F···Cl2	0.91	2.31	3.187 (3)	161
N4—H4B···Cl1	0.91	2.42	3.186 (3)	142
N4—H4A···Cl1^iii^	0.91	2.27	3.136 (3)	160
N4—H4C···Cl2^iv^	0.91	2.21	3.123 (2)	178

Symmetry codes: (i) *x*, *y*+1, *z*; (ii) *x*−1/2, −*y*+3/2, *z*; (iii) *x*+1/2, −*y*+1/2, *z*; (iv) *x*, *y*−1, *z*.
